# A technique to determine the fastest age-adjusted masters marathon world records

**DOI:** 10.1186/s40064-016-3190-5

**Published:** 2016-09-08

**Authors:** Paul M. Vanderburgh

**Affiliations:** Office of Graduate Academic Affairs, University of Dayton, 300 College Park, Dayton, OH 45469-1620 USA

**Keywords:** Aging, Physical performance, Older adults, Distance running, Handicap

## Abstract

**Introduction/Purpose:**

This study’s purpose was to develop and employ a technique to determine the fastest masters marathon world records (WR), ages 35–79 years, adjusted for age (WRadj).

**Methods:**

From single-age WR data, a best-fit polynomial curve (WRpred1) was developed for the larger age range of 29–80 years for women and 30–80 years for men to improve curve stability in the 35–79 years range. Due to the relatively large degree of data scatter about the curve and the resultant age bias in favor of older runners, a subsample was constituted consisting of those with the lowest WR/WRpred1 ratio within each five-year age group (N = 11). A new polynomial best-fit curve (WRpred2) was developed from this subsample to become the standard against which WR would be compared across age. WRadj was computed from WR/WRpred2 for all runners, 35–79 years, from which the top ten fastest were then determined.

**Results:**

The WRpred2 model reduced data scatter and eliminated the age bias. Tatyana Pozdniakova, 50 years, WR = 2:31:05, WRadj = 2:12:40; and Ed Whitlock, 73 years, WR = 2:54:48, WRadj = 1:59:57, had the fastest WRadj for women and men, respectively.

**Conclusions:**

This technique of iterative curve-fitting may be an optimal way of determining the fastest masters WRadj and may also be useful in better understanding the upper limits of human performance by age.

## Background

Marathon world records (WR) have been officially recorded for each integer age from 5 to 92 years for women and 5 to 93 years for men (http://www.arrs.net/SA_Mara.htm). Characteristics of the age versus WR plot for this age range have been assessed by 5 (Lara et al. 2014) and 1 year (Knechtle et al. 2014) age intervals; both studies confirming the describing of U-shaped plots. Despite the fact that the aging process has an inevitable slowing effect on distance run velocity (Lepers and Cattagni 2012; Reaburn and Dascombe 2008), age-adjusted comparisons (i.e., the influence of age factored out of each WR) have not been published via peer review.

Age adjustments have been developed for the 5 km (5 K) run (Vanderburgh and Laubach 2007) and the marathon (Vanderburgh 2015a), both of which also included a body weight adjustment thus enabling performance comparisons between individuals of different age and body weight within sex. The 5 K model was later validated for recreational runners by Crecelius et al. (2008) who also controlled for body composition and effort. Its age adjustment was developed from the empirically based linear relationship between age and VO_2peak_, controlling for the confounding effects of body composition and self-reported physical activity (Jackson et al. 1995, 1996). The marathon model’s age adjustment, validated empirically (Vanderburgh 2015a), was based on deviations from age group WR, a technique also used to examine the validity of the popular Boston Marathon qualifying times (Vanderburgh 2013).

Determination of the fastest age-adjusted masters marathon WR (WRadj) also relies on age adjustments. The linear technique used in the 5 K model (Vanderburgh and Laubach 2007) is incongruent with the age versus WR curvilinear trend for marathon runners between 18 and 80 years (Vanderburgh 2015a). Furthermore, there are no published data for the correlation between VO_2peak_ and the marathon that include older runners. While the age adjustment used in the marathon model is based on the age versus WR best-fit curve, use of this as a standard to compare WR across age is problematic given the high degree of scatter about the curve especially in the older age groups (Vanderburgh 2015b) which would virtually guarantee that the fastest WRadj would be among the oldest runners (further explained in the “Methods” section).

Vanderburgh (2015b) examined a double-iterative method to reduce such age-related scatter for WR marathon holders. It essentially involved deleting all data points above the best-fit age versus WR curve (the slowest age-adjusted WR), re-fitting the best-fit polynomial, then again deleting those above the curve to generate the final curve from which WR comparisons were made. The limitation of this method was that resultant data points, from which the standard best-fit curve would be developed, were unevenly distributed across the age range. This left large age gaps within which there were no WR performances (e.g., ranges with no data points after the second iteration: women: 29–47 years and men: 42–65 years). This would likely contribute to inaccuracies in the shape of an optimal best-fit polynomial curve.

Age-adjusted performances have been popularized by the World Masters Athletics organization (WMA), whose calculators are used to compare performances of multiple track and field events and distance runs, including the marathon (http://www.world-masters-athletics.org/laws-a-rules/appendixes-and-tables). Official WMA calculators are found at http://www.howardgrubb.co.uk/athletics/wmalookup15.html [cited 2 Jan 2016]. Derivations of the official standards are found at http://www.runscore.com/Alan/AgeGrade.html [cited 2 Jan 2016]. Though approved by the WMA for event-wide use these standards have not been validated via published peer-review. While current age group WR performances were used to generate the curve-fitting that determines the WMA age standards, two potential methodological limitations are relevant.

First, the standard was formed by forcing the age versus age-standard marathon time curve to be faster at every age than the actual age-based WR. Age standard was defined as “what is believed to be the fastest possible time someone of that age can run for that distance.” For example, the WMA age-standard for a woman of 63 years is 3:03:50; the actual world record at this age is 3:07:48. No explanation was offered regarding the precise determination of the age standard. Second, two women’s WR for the ages of 49 and 50 were deleted because they appeared “too fast.” No operational definition was provided for the threshold above which “too fast” could be ascertained.

Another method that might be considered for such modeling is the “convex hull” algorithm (http://mathworld.wolfram.com/ConvexHull.html [cited 2016 Aug 9]), which for this application, is essentially a method to determine the lowest (i.e., fastest WR) points in the age versus WR scatterplot. If each scatterplot point were a pin in a board, and a rubber band were stretched around the entire scatterplot and pulled toward to the top of the scatterplot, the pins that the rubber band touched would represent the lowest and, hence, the fastest WR. There are two limitations with this method, however. First, the model must include the youngest and the oldest age points, regardless of whether they are among the fastest WR. Second, the convex hull method can be unduly influenced by outliers which would contribute to large space within which no data points appear (analogous to the rubber band being “pulled” lower by an exceptional WR). This would contribute to a loss of precision regarding the shape of the resultant curve and an overestimation of the influence of outlier points.

Therefore, a more optimal method of determining the fastest WRadj was warranted. Such a technique should be empirically based on deviation from a best-fit age versus WR curve essentially free of the data scatter especially prominent among older runners and the scatterplot gaps at certain age ranges resulting from the convex hull or double-iterative method. This study’s purpose then was to propose and employ a novel technique to determine the fastest masters (35–79 years) WRadj for men and women.

## Methods

Subjects were current masters WR holders as officially recorded by the Association of Road Racing Statisticians (http://www.arrs.net/SA_Mara.htm). Only WR on looped courses, where start and finish are in the same proximity, are indicated in this reference. As these data are in the public domain, informed consent was neither possible nor necessary. The university’s institutional review board ruled that analyses of these data were exempt from review given the public nature of the data.

Though masters runners are those 35 years and older (http://www.world-masters-athletics.org/laws-a-rules/appendixes-and-tables), the age range for which the fastest WRadj would be determined in this study was 35–79 years for both sexes. The upper limit was chosen based on recent research on aging and the marathon (Lara et al. 2014; Hunter and Stevens 2013; Ahmadyar et al. 2015) which used the same upper limit of 79 years due to very low participation rates at 80 years and older. Indeed, in the 2014 New York City Marathon (total number of finishers = 50,530), there were two women and 10 men 80 years or older (http://web2.nyrrc.org/cgi-bin/start.cgi/mar-programs/archive/archive_search.html). In the 2014 Chicago Marathon (total number of finishers = 40,602), there were no women and three men 80 years or older (http://results.chicagomarathon.com/2014/). For determination of the standard curve, however, the 80 years WR of both sexes were added to contribute to a more valid estimate of the best-fit curve’s shape especially at the oldest extreme. This was deemed appropriate as the 80 years WR were notably faster than those at 78 and 79 years. Importantly, and clearly a judgment call, WR above 80 years were not included given the fact that the corresponding WR would be substantial outliers, especially for women (http://www.arrs.net/SA_Mara.htm).

Similarly, to contribute to the accuracy of the best-fit standard curve’s shape at the youngest masters 35–39 age group, 29–34 and 30–34 age groups were added to the women’s and men’s samples, respectively. The youngest age of 29 and 30 years corresponded to the ages of the open WR holders. In short, the sample from which the standard curves were developed would include 11 age groups but the sample from which WRadj was determined was comprised of nine age groups, specifically 35–39, 40–44, 45–49, 50–54, 55–59, 60–64, 65–69, 70–74, and 75–79 years.

Age versus WR scatterplots were generated and best-fit 2nd order polynomial prediction curves, WRpred1, were determined for each. Fundamentally, such curves become the standard by which WRadj can be evaluated. Points above the line would be considered “age-adjusted slower” because they are slower than that predicted by the curve. Points below the line, then, would represent the fastest age-adjusted times. Furthermore, the ratio of WR/WRpred, essentially the deviation from the expected WR at a particular age, would be an indication of how far below the curve, thus allowing precise comparisons of age-adjusted performance.

In this case, based on previous age versus WR plots for men and women, 35–80 years (Vanderburgh 2015a), both scatterplots were expected to demonstrate a high degree of scatter above and below the WRpred1 curves among the older runners. This scatter would present an important limitation: since deviation below the WRpred1 curve denotes the fastest WRadj and would be largest among the older runners, then the fastest WRadj would be among the oldest WR holders. Because of the scatter above the curve, the slowest WRadj would also come from the oldest WR holders. In short, the best-fit curve, WRpred1, imposes a bias against younger WR holders, who would have virtually no chance of having the fastest WRadj.

To mitigate this age bias, an iterative process of curve-fitting was employed. Specifically, a new subsample of WR holders was constituted, corresponding to those with the lowest WR/WRpred1 ratio within each of the 11 age groups. A new age versus WR scatterplot from the subsample (N = 11) was used to form a new best-fit curve, WRpred2. Conceptually, this new curve would be a more accurate and precise representation of the “true” age versus WR relationship as it consists of only the fastest age-adjusted WR performances. Noteworthy was the selection of the lowest WR/WRpred1 ratios, not the fastest WR within each age group to develop the new subsample. This was because the metric for fastest WRadj was, in fact, the WR/WRpred1 ratio, not fastest WR.

Finally, WRadj was calculated as the WR/WRpred2 ratio for each masters WR holder, 35–79 years, multiplied by the WRpred2 for the open WR holder of each sex. Elimination of age bias in favor of older runners was determined by comparison of the Pearson correlation coefficients for age versus WR/WRpred1 and age versus WR/WRpred2. A rank order indicated the top ten fastest WRadj in each sex. Operationally, then, WRadj was the percent deviation from the predicted WR curve (WRpred2) multiplied by the predicted open WR. WRadj could be interpreted, then, as the actual time a WR holder, based on his/her current WR, would achieve if he/she “turned back the clock” to the age of the open WR holder, 29 years for women and 30 years for men (http://www.arrs.net/SA_Mara.htm).

## Results

**Fig. 1:**
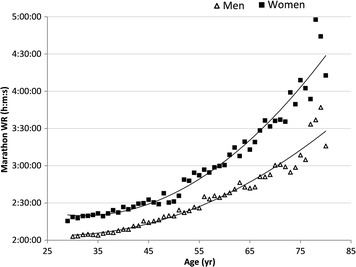
Marathon world records by age. Best-fit curves (WRpred) are 2nd order polynomials

In Fig. 1, the scatterplots of age versus WR for the 11 age groups, data points exhibited the expected scatter above and below the best-fit curve (WRpred1) particularly among the oldest runners. Since largest percent distance below the curve corresponded to the fastest WRadj then visual inspection of Fig. 1 suggested a bias in favor of older younger runners in the determination of the fastest WRadj for either sex. Pearson correlation coefficients of age versus WR/WRpred1 for the 20 lowest ratios of each sex confirmed the bias (r = 0.56 for women and 0.53 for men, *p* < 0.01 for both). The same correlation coefficients for age versus WR/WRpred2, however, suggested reduction of the bias (r = 0.09 for women and 0.33 for men, *p* > 0.10 for both). The 20 lowest ratios were used because the scatter below the curve is where increasing age would be associated with faster WRadj in WRpred1—where the bias matters (i.e., bias amongst the slowest WRadj is inconsequential). Above the curve, where the slower WRadj holders are, one would expect WRadj would be slower with increasing age. To include all WR holders would cancel out the correlations to essentially zero. As expected, the subsample’s WRpred2 fit improved from WRpred1 (R^2^ = 0.990 vs. 0.916 for women and 0.992 vs. 0.901 for men, *p* < 0.001 for both). The equations for WRpred2 were:

1 $$\begin{aligned} {\text{Women}}&{:}\,{\text{WRpred2}} = 0.0.0000308562\, \times \,{\text{Age}}^{2} - 0.0018125288 \\ & \quad \times \,{\text{Age}}\, + \,0.121203508 \\ \end{aligned}$$

2 $$\begin{aligned} {\text{Men:}}\,{\text{WRpred2}} & = 0.0000137573\, \times \,{\text{Age}}^{2} - 0.0005140915 \\ & \quad \times \,{\text{Age}}\, + \,0.0879275237 \\ \end{aligned}$$

The large number of decimal places was necessary for the precision of the WRpred2 curves, especially with the characteristic exponents. As expected, due to the mandatory selection of the lowest WR/WRpred1 values within each age group, the largest age gaps with no data points were 9 years, each occurring only once within each sex.

**Fig. 2:**
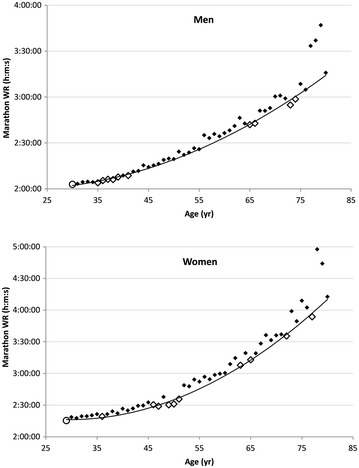
Best-fit resultant curves from the subsample (N = 11) of lowest WR/WRpred2 ratio (and, therefore, fastest WRadj) superimposed over the Fig. 1 *scatterplot* for each sex. The top ten WRadj marathoners within the 35–79 age range are indicated by *open diamonds*. The open WR for each sex is indicated by *open circles*

WRadj was calculated for each WR holder 35–79 years, using the open WRpred2 of 2:16:13 for women and 2:02:14 for men multiplied by WR/WRpred2. Figure 2 shows the resultant best-fit curve, WRpred2, superimposed over the scatterplot of age versus WR for each marathoner in the 11 age groups. Data points corresponding to the lowest “altitude” relative to the standard curve are those with the fastest WRadj times. Therefore, Tatyana Pozdniakova (50 years, WR = 2:31:05, WRadj = 2:12:40) and Ed Whitlock (73 years, WR = 2:54:48, WRadj = 1:59:57) are the fastest WRadj holders of all time for women and men, respectively. The resulting top ten WRadj of all time for the 35–79 years masters age range are labeled within Fig. 2 and detailed in Table 1.

**Table 1:** Fastest age-adjusted marathon world records (WRadj)

Rank	Name	Date set	Location	Age	Actual	WR/WRpred2	WRadj
*Women*							
1	Tatyana Pozdniakova	6-Mar-05	Los Angeles, USA	50	2:31:05	0.9740193	2:12:40
2	Tatyana Pozdniakova	7-Mar-04	Los Angeles, USA	49	2:30:17	0.9801653	2:13:30
3	Yoko Nakano	23-Nov-12	Otawara, JPN	77	3:53:42	0.9860649	2:14:19
4	Tatyana Pozdniakova	19-Mar-06	Los Angeles, USA	51	2:35:46	0.9922014	2:15:09
5	Tatyana Pozdniakova	13-Oct-02	Providence, USA	47	2:29:00	0.9931888	2:15:17
6	Helga Miketta	13-Oct-13	Essen, DEU	72	3:35:29	0.9932380	2:15:17
7	Emmi Lüthi	26-Apr-09	Zurich, CHE	65	3:12:57	1.0017334	2:16:27
8	Emmi Lüthi	28-Oct-07	Luzern, CHE	63	3:07:48	1.0072151	2:17:12
9	Irina Mikitenko	28-Sep-08	Berlin, DEU	36	2:19:18	1.0083242	2:17:21
10	Tatyana Pozdniakova	3-Mar-02	Los Angeles, USA	46	2:30:26	1.0130790	2:17:59
*Men*							
1	Ed Whitlock	26-Sep-04	Toronto, CN	73	2:54:48	0.9812516	1:59:57
2	Ed Whitlock	10-Apr-05	Rotterdam, NED	74	2:58:40	0.9908487	2:01:07
3	Haile Gebreselasie	28-Sep-08	Berlin, DEU	35	2:03:58	0.9919445	2:01:15
4	Mariko Kipchumba	21-Oct-12	Reims, FR	38	2:06:05	0.9920711	2:01:16
5	Clive Davies	13-Sep-81	Eugene, USA	66	2:42:49	0.9924742	2:01:19
6	Kenneth Mungara	5-Jul-15	Gold Coast, AUS	41	2:08:42	0.9933218	2:01:25
7	Derek Turnbull	12-Apr-92	London, ENG	65	2:41:57	0.9984809	2:02:03
8	Jaouad Gharib	26-Apr-09	London, ENG	36	2:05:27	0.9984906	2:02:03
9	Jaouad Gharib	22-Apr-12	London, ENG	39	2:07:44	0.9988831	2:02:06
10	Mark Kosgei	27-Oct-13	Frankfurt, DEU	37	2:06:16	0.9993758	2:02:10

## Discussion

This best-fit iterative method demonstrates a statistically sound and replicable technique to determine not only the fastest WRadj but to rank order all WR holders by WRadj as well. The key innovation is determining WRadj using a standard curve that more closely conforms to the upper limits of performance among master’s runners by using only the fastest WR/WRpred1 ratios within each age group. Figure 2 provides a graphic representation of the effects of the technique: the revised best-fit curve shifts down and to the right thus capturing the “fastest of the fastest” age-adjusted marathoners. With one data point for each age group constituting the scatterplot, the resulting best-fit curve is less likely to be influenced by outliers than the convex hull method.

As expected, the convex hull method, applied to the present data, yielded seven data points for each sex, which contributed to large spaces between certain points: 14 and 15 years for women and 11 and 14 years for men. In terms of ordinal ranking of WRadj for women, both methods included the three of the same marathoners but the rankings were different for each ranking, 1st through 5th. For men, both methods shared the same four marathoners but rankings were different for two places. Furthermore, an example difference for each sex between the two models was calculated. For women, the top-ranked WRadj woman (50 years) in the present iterative model, was 99 s faster than the second place woman (77 years), whereas the convex hull indicated that the 77 years woman was 6 s faster than the 50 years woman—an overall difference of 105 s. The first and second-ranked WRadj man (73 and 35 years in both methods) had an advantage of 63 s with the convex hull model and 80 s with the present model—a difference of 17 s. From these analyses, one cannot conclude that one model is more valid than the other. However, because the present iterative method included all seven of the convex hull data points and four additional for each sex, one might conclude that the present model exhibited more precision and less effects of outliers than that of the convex hull.

Another relevant characteristic of this iterative methodology was that the key metric for WRadj was a ratio of WR/WRpred2. This meant that the selection of the 11 subjects of the subsample was also based on the same corresponding ratio, WR/WRpred1, not the fastest WR, within each masters age category. The importance of this distinction can be illustrated with an example. Among women 45–49 years, the fastest WR was 2:29:08 (45 years, WR/WRpred1 ratio = 1.0234) yet the lowest ratio in that age group corresponded to a 49 years woman whose WR was 2:35:49 (WR/WRpred1ratio = 0.9644). The 45 years woman’s ratio indicated that her WR was actually slower than that predicted by the WRpred1 curve. In fact, the 49 years old woman actually earned second place among all women WR holders for her WRadj of 2:13:30, compared with that of the 45 years runner, 2:21:22.

Another advantage of this technique was that it selected the fastest among the entire sample of WR holders from ages 35–79, not just from the subsample of fastest WRadj within each age category. To do the latter would have omitted the women’s 4th and 5th and the men’s 2nd, 8th, 9th and 10th place finishers from Table 1. The only utility of the subsample was to establish WRpred2, the “bar” against which all WR holders would be evaluated.

Perhaps peculiar is the finding that the slope of the curve is quite sensitive to age differences. For example, Tatyana Pozdniakova’s 1st place (Table 1) 2:31:05 at age 50 actually shows a faster WRadj than that of her 2:30:17 performance at age 49. In this case, 1 year of age difference contributed to an *actual* difference of 48 s but a *predicted* difference of 108 s. Therefore, her performance at 50 years yielded a lower WR/WRpred ratio—the very ratio used to rank WRadj among WR holders. Figure 2 depicts this phenomenon in that the data point at 50 years is slightly lower below the line than that of 49 years.

A useful characteristic of the WRpred2 standard curve (Eq. 1) is that it can be used to compare any masters marathon performances of recreational runners. Instead of using WR, one can use actual marathon run time (MRT) to compute the ratio, MRT/WRpred2, with smaller number indicating faster age-adjusted performance. Furthermore, unlike the ratio employed in the WMA standards, the present method is empirically and algorithmically determined, not by a best-guess method upon which WMA standards appear to be based.

Since the common unit of measure for both sexes is variance from the predicted WR, the WR/WRpred2 ratio can be used to compare male with female WR performances. Important to note is the fact that the WR/WRpred2 ratio is appropriate for between-sex comparison, not WRadj. The latter factors in the predicted open WR within each sex. In the present study, for example, Tatyana Pozdniakova (50 years, WR = 2:31:05, WRadj = 2:12:40), the fastest WRadj for women; had a smaller ratio than Ed Whitlock, her male counterpart (73 years, WR = 2:54:48, WRadj = 1:59:57), at 0.9740 and 0.9813, respectively. This suggests that Ms. Pozdniakova has the fastest age-adjusted marathon performance of all time. Within sex, however, WRadj provides a more meaningful or perhaps useful score than the WR/WRpred ratio alone as the h:m:s units of WRadj are interpretable by virtually any runner. As stated previously, it is a statistical estimate of what the WR holder would run if he/she were of the age of the open WR holder for that sex. Such an estimate has been published elsewhere (Vanderburgh and Laubach 2007; Vanderburgh 2015a).

Use of the present iterative technique to examine elite age-adjusted performances may also inform the study of the effect of aging on physical performance in a way that controls for confounding factors such as physical activity level, body composition, effort, etc. In other words, this technique may yield important information about the inevitable loss of function with age, or the physiological limits of human performance with age.

## Conclusions

To date, no published evidence has identified the fastest age-adjusted marathons of all time for masters men and women. The best-fit iterative method used here, which reduces data scatter and scatterplot age gaps, leads to an assessment standard that may be the fairest way to determine the fastest WRadj. Applied to all world record holders for each age from 35–79 years, the results indicated that Tatyana Pozdniakova (50 years, WR = 2:31:05, WRadj = 2:12:40) and Ed Whitlock (73 years, WR = 2:54:48, WRadj = 1:59:57) are the fastest age-adjusted masters marathoners. This technique may also be helpful in examining the independent effects of age on the upper limits of human performance.
